# Are Owls and Larks Different When it Comes to Aggression? Genetics, Neurobiology, and Behavior

**DOI:** 10.3389/fnbeh.2020.00039

**Published:** 2020-03-17

**Authors:** Scott H. Deibel, Robert J. McDonald, Nathan J. Kolla

**Affiliations:** ^1^Department of Psychology, Memorial University of Newfoundland, St. John’s, NL, Canada; ^2^Department of Neuroscience, University of Lethbridge, Lethbridge, AL, Canada; ^3^Waypoint Centre for Mental Health Care, Penetanguishene, ON, Canada; ^4^Centre for Addiction and Mental Health, Toronto, ON, Canada; ^5^Department of Psychiatry, University of Toronto, Toronto, ON, Canada

**Keywords:** circadian rhythms, aggression, circadian misalignment, chronotype, genetics, neurobiology, behavior

## Abstract

This review focuses on the contribution of circadian rhythms to aggression with a multifaceted approach incorporating genetics, neural networks, and behavior. We explore the hypothesis that chronic circadian misalignment is contributing to increased aggression. Genes involved in both circadian rhythms and aggression are discussed as a possible mechanism for increased aggression that might be elicited by circadian misalignment. We then discuss the neural networks underlying aggression and how dysregulation in the interaction of these networks evoked by circadian rhythm misalignment could contribute to aggression. The last section of this review will present recent human correlational data demonstrating the association between chronotype and/or circadian misalignment with aggression. With circadian rhythms and aggression being a burgeoning area of study, we hope that this review initiates more interest in this promising and topical area.

## Introduction

In chronobiology, chronotype refers to one’s sleep-wake timing (Roenneberg, 2012). Genetics, age, gender, and environmental stimuli contribute to the expression of chronotype (Barclay et al., 2013; Foster et al., 2013; Wicht et al., 2014). Recent evidence suggests that the colloquial statement “the early bird gets the worm” holds some weight as evening preference is associated with a higher incidence of disease (Roenneberg and Merrow, 2016). Human aggression has a sizable impact on society with exorbitant resources allocated to its reduction or aftermath. Recent research suggests that the use of aggression is higher in evening types, with several studies suggesting that traits involved in pre-meditated aggression are also associated with a late chronotype (Jonason et al., 2013; Schlarb et al., 2014; Rahafar et al., 2017). The deleterious effects associated with an evening preference could be the simple result of experiencing chronic, but mild circadian rhythm disruption.

This work is a scoping/narrative review that will discuss the relationship between circadian rhythms and aggression, with a broad focus that encompasses genetics, neural circuitry, and human behavior. The following keywords were entered in various combinations into Google Scholar in December 2018 to inform our review: chronotype, eveningness, psychopathy, antisocial personality disorder (ASPD), aggression, reactive aggression, proactive aggression, circadian rhythms, neurobiology, genetics, polymorphisms, rodents, and humans.

The tenet of this article is that circadian rhythm dysfunction contributes to aggression. To guide this supposition, we draw from the wealth of genetic association studies, animal models of circadian rhythms and aggression, and human correlational data. The first section of this article will discuss circadian rhythm misalignment and aggression. The second section of this article will focus on several genes implicated in circadian rhythms that could contribute to aggressive behavior. The third section of the article will outline how the neural networks underlying forms of aggression are similar to those involved in learning and memory and how the dysfunction present in both aggression and circadian rhythm disruption involves changes in the interactions of these networks. Finally, we will discuss recent human correlational data, which demonstrates that chronotype and/or circadian misalignment are associated with aggression.

## Section 1: Circadian Misalignment and Aggression

### Circadian Rhythm Misalignment

To better take advantage of the environment, behavior and physiological processes need to occur at the appropriate time. Circadian rhythms can be thought of as our internal timekeepers that regulate oscillating behavior and physiology that takes approximately 24 h to complete one revolution. Body temperature, hormone secretion, metabolism, and the timing of sleep, activity, and meals are other processes that are modulated by circadian rhythms (Barnard and Nolan, 2008; Zelinski et al., 2014a; Roenneberg and Merrow, 2016). In order to predict events, circadian rhythms need to be synchronized with the environment. Light is the strongest entraining cue (zeitgeber), but food, exercise, social interaction, drugs, temperature, and learning can entrain circadian rhythms in certain circumstances (Merrow et al., 2005; Ralph et al., 2013; Krishnan and Lyons, 2015).

If the goal of circadian rhythms is to predict environmental events, what happens when there is a discrepancy between internal and external time? As eloquently put by Roenneberg and Merrow (Roenneberg and Merrow, 2016): “Without entrainment, the system loses its main advantage—faithfully predicting the regular changes of its environment (pg. R432).” They further highlight the gravity of this situation by positing that inaccurate circadian predictions are perhaps more detrimental than if there were no circadian predictions at all (Roenneberg and Merrow, 2016). It can be thought of as a sub-optimal form of entrainment (Roenneberg and Merrow, 2016). Circadian misalignment, in the simplest sense, occurs when we are active when we should be sleeping. It can present in its mildest form as waking up earlier with an alarm clock or more seriously as working throughout the night. Readers are encouraged to consult the excellent review by Baron and Reid (2015) that outlines circadian misalignment and its impact on health and disease.

Circadian rhythm misalignment has many deleterious effects on human health (Barnard and Nolan, 2008; Escobar et al., 2011; Zelinski et al., 2014a). Major problems that affect virtually all aspects of physiology arise when circadian rhythms are unsynchronized from the environment (Kuhn, 2001; Zelinski et al., 2014a). Chronic circadian rhythm misalignment has been associated with cognitive impairments, heart disease, metabolic syndrome, shortened lifespan, and cancer (Haus and Smolensky, 2006; Zelinski et al., 2014a; Banks et al., 2016). Artificial light at night is purported to be the major mechanism for the harmful effects of circadian misalignment on the brain and the body (Navara and Nelson, 2007; Bedrosian and Nelson, 2017).

While approximately 20% of the workforce in industrialized countries engage in shift work (Baron and Reid, 2015), most people are likely experiencing a milder yet chronic state of circadian rhythm disruption. It is thought that in some cases circadian misalignment in society is a result of one’s preferred timing of their sleep-wake rhythm (Baron and Reid, 2015). This Interindividual variation in the phase of entrainment is referred to as *chronotype* (Horne and Östberg, 1976; Roenneberg et al., 2003, 2004; Levandovski et al., 2013; Roenneberg and Merrow, 2016). Although chronotype exists on a continuum, in the extremes of the distribution, there are people who wake and go to sleep early (*larks*; morning types) and others who do so later (owls; evening types; Roenneberg, 2012; Levandovski et al., 2013; Roenneberg and Merrow, 2016). There are also intermediate (neither morning or evening type), which creates three chronotype classifications (Roenneberg et al., 2004; Adan et al., 2012). Neither or intermediate type is the most common in the population (60%), with the remaining being either morning (20%) or evening types (20%; Adan et al., 2012).

Misalignment could be a result of a mismatch between a person’s natural sleeping preference and the schedule dictated by his or her work and social habits (Baron and Reid, 2015). Some studies have indeed found that in late chronotypes, there is circadian misalignment among sleep and markers of phase (Kerkhof and Lancel, 1991; Duffy et al., 1999; Mongrain et al., 2006). While sleep duration does not always correlate with chronotype (Horne and Östberg, 1976; Robilliard et al., 2002; Randler and Vollmer, 2013), as discussed below, sleep duration can vary during the week and weekend in a chronotype dependent manner (Wittmann et al., 2006; Adan et al., 2012; Randler et al., 2017). This misalignment puts individuals in a continuous state of catch-up, or chronic circadian misalignment, and is referred to as *social jetlag* (Wittmann et al., 2006). While an evening chronotype does not always incur social jetlag, it is more common in evening chronotypes (Wittmann et al., 2006; Baron and Reid, 2015; Roenneberg and Merrow, 2016). In other words, social jetlag can be thought of as a discrepancy between an individual’s work schedule and his or her endogenous clock (Baron and Reid, 2015). Strikingly, 87% of day workers (65,000 people located in central Europe) experienced social jetlag (Roenneberg et al., 2012). Social jetlag is a less severe form of shift work, but nonetheless a form of chronic circadian misalignment (Wittmann et al., 2006; Roenneberg and Merrow, 2016).

### Aggressive Behavior

Aggression is a heterogeneous group of behaviors, but it is broadly defined as any behavior that threatens to, or does harm/injure others (Berkowitz, 1993; Baron and Richardson, 2004). It is an adaptive behavior if displayed in the appropriate context; however, if exaggerated and/or it often occurs in inappropriate contexts, it is considered maladaptive (Bedrosian and Nelson, 2018). Aggressive behavior is found in virtually all species, with some species being more aggressive than others. Aggression has a major impact on society; in addition to concerns of safety, significant resources are allocated to curtail or ameliorate the aftermaths of aggression (Temcheff et al., 2011; Waltes et al., 2016).

From a psychiatric perspective, there are several disorders in The Diagnostic and Statistical Manual of Mental Disorders Version 5 (American Psychiatric Association, 2013), that strongly feature aggression, such as conduct disorder, oppositional defiant disorder, intermittent explosive disorder, ASPD and borderline personality disorder. However, as highlighted by van Donkelaar (2018), categorical diagnoses and featured comorbidities, such as aggression, are likely extremes in a normally distributed continuum of the behavior in question rather than binary constructs.

ASPD, which we discuss throughout this review, is characterized by impulsivity and high levels of aggression, with a prevalence of approximately 7% in the community and 50% in the penal system (Swanson et al., 1994; Fazel and Danesh, 2002; Coid et al., 2006). ASPD presents initially as conduct disorder during youth. Hence, adults with ASPD are likely to have had other externalizing conditions as children or adolescents (Reef et al., 2011; Waltes et al., 2016).

Psychopathy is related to ASPD but differs in several key ways. In addition to antisocial behavior, psychopathy includes impaired emotional responses such as decreased empathy, remorse and guilt (Hare, 1996). Individuals with psychopathy are ego-driven and do not abide by social norms (Hare, 1996; Blair, 2003). Defiance of social norms is thought to be mediated by impaired emotional recognition in others, the inability to recognize that behavior has aversive consequences, and the failure to stop behaviors that have elicited negative consequences in the past (Blair, 2003). In terms of prevalence, it is estimated that 1% of the general, and 15–25% of the penal population endorse psychopathy (Hare, 1996). One-third of those with ASPD meet the criteria for psychopathy (Hart and Hare, 1996; Blair, 2003).

While debated, it is generally assumed that humans are one of the more aggressive species (Waltes et al., 2016; Wrangham, 2018). The contention regarding this statement might be due to the fact that aggression is a multi-faceted construct (Wrangham, 2018). There are many different terms and classification systems for the different kinds of aggression, but they can generally be differentiated into two broad categories: proactive (pre-meditated) and reactive (impulsive; Waltes et al., 2016; Wrangham, 2018). One’s motivation for engaging in aggression is the key distinguisher between these two types of aggression (Raine et al., 2006). Proactive aggression is planned aggression that is executed when one judges that he or she is likely to achieve an extrinsic or intrinsic goal (Wrangham, 2018). Bullying, stalking, and premeditated homicide are an example of proactive aggression (Wrangham, 2018). Proactive aggressors are more likely to be recidivists and endorse psychopathy (Kockler et al., 2006; Stanford et al., 2008; Swogger et al., 2015; Wrangham, 2018). In comparison, reactive aggression is executed to remove an aversive stimulus and is often driven by frustration or eruption of anger (Wrangham, 2018). Crimes of passion and bar fights prompted by intoxication are examples of reactive aggression (Wrangham, 2018). Along these lines, emotional arousal further distinguishes the two types of aggression, with proactive aggression involving little emotional arousal and reactive aggression involving more (Raine et al., 2006; Wrangham, 2018). Anger is always associated with reactive aggression (Wrangham, 2018). In addition to behavior, these different types of aggression are thought to have disparate underlying neurobiologies and neurocognition (van Donkelaar et al., 2020). These constructs also have convergent properties as they are very highly intercorrelated and are often both present in populations with pathologies that feature aggression, such as antisocial and borderline personality disorders (Gardner et al., 2012; Lobbestael et al., 2013; Rosell and Siever, 2015).

Aggressive behavior can be distinguished by the motivating factors and goals for its execution. Like chronotype, aggressive behavior is thought to exist on a continuum and is thought to be normally distributed in the population (Roenneberg, 2012; Levandovski et al., 2013; Walters and Ruscio, 2013; Roenneberg and Merrow, 2016; Waltes et al., 2016; van Donkelaar, 2018). It is important to elucidate the etiology of aggression, with the hope of reducing its prevalence. Circadian misalignment is one hypothetical mechanism for aggressive behavior. Although this hypothesis is largely untested, the next discussion will provide tangential evidence suggesting that circadian misalignment contributes to the manifestation of aggressive behavior.

### Circadian Misalignment Is a Possible Mechanism for Aggression

While most studies that have reported an effect of chronotype in relation to health and disease name circadian misalignment as a likely mechanism, these findings are rarely confirmed (Baron and Reid, 2015). To the best of our knowledge, no study has investigated whether aggression is accompanied by objective measures of circadian rhythm misalignment. In theory, the notion that circadian misalignment elicits increased aggression would rely on diurnal differences in aggression. Chronotype differences in aggression have been proposed as evidence that circadian fluctuations influence aggression (Hood and Amir, 2018). The presence of increased verbal and physical aggression in the afternoon in Alzheimer’s disease patients has also been suggested as one explanation for diurnal differences in aggression (Coogan et al., 2013; Hood and Amir, 2018). However, until now, there has never been a demonstration that aggression is influenced by an endogenously-generated circadian rhythm (Hood and Amir, 2018). For the first time, Todd et al. (2018) demonstrated in mice that there is a true circadian rhythm in territorial aggression that persists under constant conditions (without zeitgebers). The resident-intruder model of reactive aggression was used to measure aggression. In this model, a rodent is singly housed for a month, which produces territorial aggression when a conspecific is introduced to their home cage. The authors concluded that either direct suprachiasmatic nucleus (master pacemaker in the brain; SCN) outputs or indirect ones *via* the subparaventricular zone influence the ventromedial hypothalamus’s modulation of aggression in a circadian manner (Todd et al., 2018). If aggression is under circadian control, it is, therefore, possible that circadian misalignment could increase the likelihood of aggressive behavior.

Several factors suggest that circadian misalignment is a possible mechanism underlying the effects of a late chronotype on aggression. First, as mentioned above, in the few studies that have assessed this line of research, a late chronotype is associated with circadian misalignment of rhythms such as melatonin release and body temperature during sleep (Kerkhof and Lancel, 1991; Duffy et al., 1999; Mongrain et al., 2006).

Second, there is a large corpus of literature linking self-reported sleep disruption or poor sleep quality with increased aggression (Kamphuis et al., 2012; Krizan and Herlache, 2016). For example, sleep disruption is a predictor of aggression, as measured by the observer-scale: The Social Dysfunction and Aggression Scale (SDAS; Wistedt et al., 1990), in prisoners with or without psychiatric histories (Vogler et al., 2014; Meijers et al., 2015). Reduced sleep duration is also associated with increased verbal aggression and anger (Randler and Vollmer, 2013). Sleep disturbances also relate to increased psychopathic and Machiavellianism traits as measured by the Short Dark Triad questionnaire (Sabouri et al., 2016; Akram et al., 2018). Sleep is partially modulated by circadian rhythms, with the timing of sleep (sleep-wake cycle; chronotype) controlled by the SCN. Disrupted sleep and/or daytime tiredness may be one of the most significant consequences of circadian misalignment (Baron and Reid, 2015). Not surprisingly, shift workers experiencing circadian misalignment also report impacted sleep schedules (Reinberg and Ashkenazi, 2008; Saksvik-Lehouillier et al., 2015). Since late chronotype is associated with poor sleep quality, shorter sleep duration, and daytime tiredness (Giannotti et al., 2002; Robilliard et al., 2002; Taillard et al., 2003, 2004; Juda et al., 2013; Horne et al., 2019), it is possible that sleep disruption could underscore the influence of chronotype-mediated circadian misalignment on aggression.

Third, disruption of circadian rhythms in Alzheimer’s disease might also speak to the relationship between circadian misalignment and aggression. Circadian misalignment and sleep disruption are hallmarks of Alzheimer’s disease (Coogan et al., 2013; Deibel et al., 2015; Deibel and McDonald, 2017). Sundowning, which involves increased activity and agitation/aggression in the late afternoon in dementia patients (Coogan et al., 2013), could be the result of circadian misalignment since circadian dysfunction is associated with more observable verbal and physical aggression in Alzheimer’s disease patients (Etcher et al., 2012). While there are likely several pathologies leading to agitation/aggression in Alzheimer’s disease, circadian rhythm misalignment seems a likely contributor.

There are a variety of factors which suggest that circadian misalignment could be a mechanism for the association between eveningness and increased aggression. However, to our knowledge, quantitative measures of circadian misalignment, such as melatonin rhythmicity have not been assessed in relation to aggression. Genetics provides another avenue to explore the link between circadian rhythms and aggression. Circadian misalignment might elicit aggression, but the mechanisms for this effect remain to be seen. Altered gene expression elicited by genetic polymorphisms and/or environmental circadian rhythm disruption could lead to increased aggressive behavior. The next section will explore recent research highlighting the putative genetic contributions to circadian rhythm generation and aggression.

## Section 2: Genes

### Genes Involved in Circadian Rhythm Generation and Aggression

Aggression and chronotype are heritable traits, with genetics explaining approximately 50% of the variance in twin studies (Koskenvuo et al., 2007; Burt, 2009; Barclay et al., 2010, 2013; Tuvblad and Baker, 2011). Non-shared environmental experiences also account for a large portion of the variance (Koskenvuo et al., 2007; Barclay et al., 2010, 2013; Veroude et al., 2016; Waltes et al., 2016).

There are a group of genes referred to as “core clock genes,” found in virtually all cell types, that generate circadian rhythmicity *via* a cell-autonomous, transcriptional-translational autoregulatory feedback loop (Reppert and Weaver, 2001; Okamura, 2004; Takahashi, 2017). The activators consist of the transcription factors Circadian Locomotor Output Cycles Kaput CLOCK [and its paralog Neuronal PAS domain protein 2 (NPAS2)] and Aryl hydrocarbon receptor nuclear translocator-like protein [BMAL1 (ARNTL); Reppert and Weaver, 2001; Okamura, 2004; Takahashi, 2017]. They form heterodimers and activate the transcription of genes encoding the following repressor proteins: PERIOD (PER1, PER2, PER3) and cryptochrome (CRY1, CRY2; Okamura, 2004; Takahashi, 2017). PER and CRY proteins then dimerize and transfer to the nucleus to inhibit the transcription of *Clock* and *Bmal1* (Reppert and Weaver, 2001; Okamura, 2004; Takahashi, 2017). Another regulatory loop influences the core loop with the nuclear receptor and transcriptional repressor REV-ERB, attenuating *Bmal1* transcription in a rhythmic fashion (Reppert and Weaver, 2001; Okamura, 2004; Takahashi, 2017). This cycle takes approximately 24 h (Takahashi, 2017).

Many studies of aggression have assessed genes that were selected for their perceived relevance in aggressive behavior. However, effect sizes tend to be small and results do not always replicate across studies. With the advent of GWAS, progress will be made in identifying genetic loci of aggressive behavior. Most candidate gene association studies of aggression have studied serotonergic (5-HT), dopaminergic (DA), and hormonal genes (Veroude et al., 2016; Waltes et al., 2016; Bedrosian and Nelson, 2018). Altered levels of 5-HT and DA have been implicated in aggressive behavior. In animals and humans, lower brain, blood, and cerebrospinal fluid levels of the 5-HT metabolite 5-hydroxy indoleacetic acid (5-HIAA) has been associated with increased aggression (Ramboz et al., 1995; Tuinier et al., 1995; Higley et al., 1996; Coccaro et al., 1998; Angoa-Pérez et al., 2012; Provençal et al., 2015; Bedrosian and Nelson, 2018). In humans, decreased DA can be associated with increased impulsive/reactive aggression (Schlüter et al., 2013; Rosell and Siever, 2015; Bedrosian and Nelson, 2018), however, the reinforcing or hedonic aspect of aggression is associated with increased DA in a region of the striatum involved in reward processing [nucleus accumbens (NA; Ferrari et al., 2003; Buckholtz et al., 2010; Bedrosian and Nelson, 2018)].

### Genetic Polymorphisms in Circadian Genes and Aggression

Genetically selecting for aggression results in a specific circadian phenotype (Benus et al., 1988). Notably, there was a longer free-running period and much longer re-entrainment to a zeitgeber change when animals were selected for aggression (Benus et al., 1988). However, it is unknown whether aggression led to altered circadian rhythms or whether an altered circadian phenotype contributed to heightened aggression. Regardless, it is likely that these differences were the result of altered clock gene expression in the SCN. The finding that *Clock* expression in the SCN and circadian expression of molecules involved in the stress response were altered in aggressive and impulsive rats supports this notion (Kerman et al., 2012). Data from transgenic mice with mutations affecting clock genes suggest that circadian dysfunction can elicit aggression (Hood and Amir, 2018). For example, knocking out the clock genes *rev-erb alpha* (Chung et al., 2014) and *clock* (Roybal et al., 2007; Coque et al., 2011) increases resident-intruder aggression, and hyperactivity/impulsivity, respectively.

Polymorphisms in circadian genes have been associated with both chronotype and aggression, especially in 5-HT and DA genes, where dysregulation is thought to mediate aggression (Tuinier et al., 1995; Higley et al., 1996; Coccaro et al., 1998; Provençal et al., 2015; da Cunha-Bang et al., 2016). For example, recent GWASs have identified loci near clock genes and those involved in the serotonergic system that highly influence morning or evening preferences (Hu et al., 2016; Jones et al., 2016; Hood and Amir, 2018). However, genetic association studies with aggression and core clock gene polymorphisms have not yet been conducted. Nonetheless, there are several genes involved or influenced by circadian rhythms that could theoretically elicit aggression when dysregulated.

Recent data have suggested that Sirtuin 1 (*Sirt1*), a gene involved in circadian rhythm generation, could also be relevant to the elicitation of aggression. *Sirt1* is a histone and protein deacetylase enzyme whose main function is to repress CLOCK- (positive activator core clock gene) mediated transcription by deacetylating promoter regions of core clock genes: BMAL1 and PER2 (Asher et al., 2008; Nakahata et al., 2008, 2009). *Sirt1* knockout mice manifest disrupted circadian rhythms (elongated free-running period), likely due to decreased CLOCK and BMAL1 proteins, and reduced* Bmal1* and *Per2* RNA in the SCN (Chang and Guarente, 2013). In humans, the minor *Sirt1* rs1467568 allele (AG or AA) has been associated with eveningness (Katzenberg et al., 1998; Kripke et al., 2008; Garaulet et al., 2012). This variant was also associated with eveningness when concomitant with the minor allele of *Clock 1131T>C* (*TC* or *CC*) single nucleotide polymorphism (SNP; Garaulet et al., 2012). In a Chinese population of male juveniles, *Sirt1* polymorphisms were associated with a predisposition for ASPD (Chang et al., 2017). Chang et al. (2017) suggest that altered SIRT1 expression disrupts circadian rhythms and that disruption contributes to the pathogenesis of ASPD. The finding that *Sirt1* polymorphisms have also been associated with a late chronotype type bolsters the argument (Garaulet et al., 2012; Chang et al., 2017).

It is possible that clock gene polymorphisms modulate genes that regulate aggression (Hood and Amir, 2018). Monoamine oxidase-A (MAO-A) catabolizes 5HT, norepinephrine (NE), and DA and is perhaps the most studied and robust candidate gene for aggressive behavior (Veroude et al., 2016; Waltes et al., 2016; Kolla and Vinette, 2017). *Mao-a* possesses a variable number tandem repeat (VNTR) that results in less (*Maoa-L*) or more efficient transcription (*Maoa-H*). However, counterintuitively, no correlation has been found between the amount of brain MAO-A and the VNTR genotypes (Fowler et al., 2007; Alia-Klein et al., 2008; Shumay et al., 2012). While most studies find that *Maoa-L* is associated with aggression, others have implicated *Maoa-H* (Kolla et al., 2014; Tiihonen et al., 2015; Veroude et al., 2016; Waltes et al., 2016; Kolla and Vinette, 2017). This effect likely depends on a variety of factors such as age, sex, concomitant conditions, and type of aggression.

Since MAO-A oscillates in brain areas, such as the ventral tegmental area (VTA) and the ventral striatum (NA), it is not surprising that clock genes are involved in its regulation (Hampp et al., 2008). It has been proposed that clock gene polymorphisms might modulate the regulatory role of MAO-A in aggression (Hood and Amir, 2018). BMAL1 and PER2 increase transcription of MAO-A, with PER2 playing an activating role (Hampp et al., 2008). In PER2 knockout mice, MAO-A expression is attenuated and fails to oscillate (Hampp et al., 2008). Due to reduced MAO-A, striatal DA is increased in these animals. SIRT1 is also involved in the activation of MAO-A *via* the deacetylation of the transcription factor Nescient Helix-Loop-Helix 2 (NHLH2; Libert et al., 2011). In contrast to PER2 knockout mice, SIRT1 knockout mice have increased 5HT, and not DA (Hampp et al., 2008; Libert et al., 2011). Correspondingly, they also show fewer behaviors consistent with anxiety and depression (Libert et al., 2011).

Besides its effect on DA *via* regulation of MAO-A, SIRT1 is also directly involved in DA synthesis. Tyrosine hydroxylase (TH) is the rate-limiting enzyme in DA synthesis. The VTA is the primary site of DA synthesis, and TH is expressed diurnally in the VTA with peak expression during the night in mice (Logan et al., 2018). While CREB is responsible for upregulating TH expression during the evening; interestingly, CLOCK inhibits TH expression during the nadir (daytime; Logan et al., 2018). The inhibiting action of CLOCK depends on SIRT1 and its cofactor nicotinamide adenine dinucleotide (NAD+; Logan et al., 2018). *Clock* knockout mice have elevated TH and increased levels of DA in the VTA. The same Clock knockout mice also exhibit increased hyperactivity and impulsivity (Roybal et al., 2007; Coque et al., 2011). Although association studies with *Clock* polymorphisms and aggression have not been performed, clock polymorphisms have been linked to other illnesses that can involve aggression, such as attention deficit hyperactivity disorder (ADHD). Disturbed sleep and symptoms typically associated with circadian misalignment, such as irritability, mild depression, daytime tiredness, and impaired cognition can also occur in ADHD (Katz et al., 2001; Baird et al., 2012; Mogavero et al., 2018).

The SIRT1 data detailed above suggest that SIRT1 polymorphisms contribute to both chronotype and aggression. SIRT1 likely influences aggression *via* its regulatory role of MAOA and subsequently DA and 5-HT expression. SIRT1 also affects DA directly *via* its effect on CLOCK, which although untested, opens up the possibility that *Clock* polymorphisms might contribute to aggression.

SIRT1 is involved in the regulation of another gene, brain-derived neurotrophic factor (BDNF), that has been implicated in mediating the effects of adverse childhood experiences on aggression. BDNF is a rhythmically expressed gene in the SCN and other brain regions, such as the hippocampus, that is implicated in cellular plasticity, neuronal survival, and morphogenesis (Liang et al., 1998; Earnest et al., 1999; Schaaf et al., 2000; Katoh-Semba et al., 2008; Rudenko and Tsai, 2014). BDNF is part of the mitogen-activated protein kinase signaling cascade, which underlies many biological processes, including regulation of circadian rhythms, learning, and memory (Eckel-Mahan and Storm, 2009; Gao et al., 2010; Rudenko and Tsai, 2014; Peixoto et al., 2015). BDNF is thought to be involved in the SCN’s response to photic input (Michel et al., 2006; Naert et al., 2006), with BDNF knockout mice showing decreased response to light pulses (Liang et al., 2000). BDNF may also modulate aggressive behavior, with exogenous BDNF eliciting proactive aggression in a highly aggressive mutant mouse strain (AKR; Naumenko et al., 2014).

Several studies suggest that the BDNF met-met polymorphism moderates the effect of adverse childhood experiences on aggression (Wagner et al., 2010; Kretschmer et al., 2012). Many of the genotypes implicated in aggression or impulsivity are often mediated by adverse environmental experiences (Caspi et al., 2002; Kendler et al., 2011; Kolla et al., 2017a). In Borderline Personality subjects, a condition that is marked by frequent anger outbursts and impulsive aggression, Wagner et al. (2010) found that those who carried the Met/Met allele of the BDNF Val^66^Met polymorphism had higher impulsive aggression scores on the Buss-Durkey-Hostility inventory (Buss and Durkee, 1957) if they had experienced childhood sexual abuse, or exposure to aggressive peers during the late stages of childhood (Wagner et al., 2010; Kretschmer et al., 2012).

SIRT1 acts as a positive regulator of BDNF by deacetylating methyl CpG binding protein 2 or by inhibiting the microRNA 134 (Gao et al., 2010). Hippocampal SIRT1 expression is affected by environmental manipulations that affect circadian rhythmicity, such as diet, sleep deprivation, shifts or inversions of the light-dark cycle, and genetic manipulation of clock genes (Chang et al., 2009; Heyward et al., 2012; Rawashdeh et al., 2014; Fang et al., 2017). BDNF is also affected by environmental manipulations that affect circadian rhythms, such as phase shifts of the light-dark cycle (Sei et al., 2003; Katoh-Semba et al., 2008). It is possible that changes in SIRT1 brain levels elicited by SIRT1 polymorphisms or environmental influences impact the manifestation of aggression *via* regulation of BDNF.

In addition to SIRT1, polymorphisms in arginine vasopressin may also influence circadian rhythms and aggression. Arginine vasopressin is a neuropeptide that modulates complex social behaviors; in the periphery, it acts as a hormone to regulate physiological processes, such as osmoregulation (Ebstein et al., 2009; Song and Albers, 2018). AVP and specifically the AVPR1a receptor (most common AVP receptor in the brain) are implicated in aggressive behavior (Ferris and Potegal, 1988; Ferris et al., 1997; Caldwell and Albers, 2004). Injecting AVP into the anterior hypothalamus can elicit aggression in hamsters, and this effect appears to be dependent on AVPR1a receptors since its antagonist reduces aggression when injected into the same area (Ferris and Potegal, 1988; Ferris et al., 1997; Caldwell and Albers, 2004). In humans, SNPs in *Avpr1a* have been associated with non-clinical childhood aggression that is assessed by parents (Pappa et al., 2016). This finding was unexpected as many candidate genes implicated in aggressive behavior were also tested: dopaminergic, serotonergic, stress, and adrenergic genes (Pappa et al., 2016).

The role of AVP in both aggression and circadian rhythms has interesting implications for how circadian rhythms might contribute to aggression. Although to our knowledge, it is unknown whether potential *Avpr1a* polymorphisms contribute to chronotype, animal data demonstrated that an increased AVP tone is associated with aggression, delayed circadian rhythms, and reduced circadian entrainability (Ferris et al., 1997; Caldwell and Albers, 2004; Yamaguchi et al., 2013). Epigenetics is a likely mechanism for an upregulated AVP tone. The *Avpr1a* gene promotor was less methylated in aggressive children, which the authors suggested was indicative of increased activity (Provençal et al., 2014). So, if AVP tone is elevated due to genetic polymorphisms or environmental factors, entrainment should be more difficult, which might create misalignment and heightened aggression.

**Figure 1:**
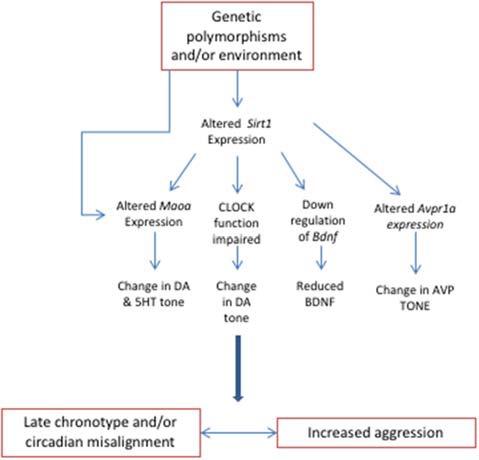
Changes in the expression of genes involved in circadian rhythms influence aggression. The environment and/or polymorphisms in genes such as *Sirt1*, *Clock*, and *Avpr1a* induce circadian misalignment, which also affects pathways that mediate aggression, such as the dopaminergic and serotonergic systems.

In summary, there are several examples of how genetic polymorphisms of genes influenced by circadian rhythms could have distal effects on aggressive behavior (Figure 1). The genotypes discussed above may be more susceptible to circadian rhythm misalignment, and these changes could contribute to the symptomology of aggression and its associated disorders. We have also highlighted that this same mechanism may be driven by environmental influences rather than genotype. It is likely that this scenario would apply to most mechanisms hypothesized to modulate aggressive behavior. In summary, we contend that changes in gene expression that are elicited *via* genotype or environmental factors act in parallel or synergistically to influence aggression. Yet, how do changes in gene expression affect the functioning and interaction of brain circuits that modulate aggressive behavior? The next section will tackle this question.

## Section 3: Brain Circuits Involved in Aggression

While altered gene expression contributes to aggressive behavior, changes in behavior will not occur unless there are downstream changes in the structure/function of the brain regions/circuits involved. As aggression is a complex behavior, many areas and circuits are involved, particularly areas that are involved in learning/memory, and emotional regulation. We will first briefly introduce the key brain regions that are involved in aggression. We will then provide a unique explanation for how aggressive behavior could be elicited by changes in the interactions of parallel learning and memory circuits. Finally, the ability of circadian rhythm disruption to perturb these brain regions, circuits and neuromodulators will also be discussed.

### Key Regions Involved

A thorough review of the regions and neural circuitry involved in aggression is beyond the scope of the present review. Readers are encouraged to refer to additional review articles for discussions of this nature (Rosell and Siever, 2015; Blair, 2016; Ling and Raine, 2018). We will briefly discuss the involvement of some regions below in aggression to provide context for how circadian rhythm disruption might act on these regions to produce aggressive behavior.

The amygdala is involved in fear and emotion recognition. Flat affect; callous, unemotional traits and lack of empathy are associated with psychopathy; amygdalar dysfunction is likely involved (Ling and Raine, 2018). Impaired amygdalar functioning leads to aberrant or failure to form stimulus-response associations, which could precipitate inappropriate behavior because the negative outcomes are not anticipated (Gruber and McDonald, 2012; Ling and Raine, 2018). Reduced amygdala volume in both forensic and community populations is strongly associated with aggression (Matthies et al., 2012; Pardini et al., 2014; Rosell and Siever, 2015; Ling and Raine, 2018). Interestingly, in violent individuals, the *Maoa* VNTR genotype affects the amygdalar surface area (Kolla et al., 2017b). These data are some of the first to demonstrate that in violent individuals, genotype contributes to morphological differences in brain regions implicated in aggression.

The ventral medial prefrontal cortex (vmPFC) and its subregions—orbitofrontal cortex (OFC), and anterior cingulate cortex (ACC)—are thought to modulate the likelihood of an aggressive response. The OFC evaluates the motivational value and affective valence of stimuli, whereas the ACC is thought to be involved in determining the action or response (Rosell and Siever, 2015). Dysregulation in the vmPFC could affect decision making, with inappropriate social outcomes likely selected (Bechara et al., 1999; Ling and Raine, 2018). Interestingly, decision making in patients with vmPFC and/or OFC damage is very similar to that of individuals with psychopathy, which commonly features proactive aggression (Koenigs et al., 2007; Ling and Raine, 2018). Psychopathy has been associated with reduced PFC total volume and reduced PFC/OFC gray matter volume (de Oliveira-Souza et al., 2008; Ermer et al., 2012; Ling and Raine, 2018).

The striatum is thought to be involved in the impulsive-antisocial and the interpersonal-affective features of aggression (Ling and Raine, 2018). The ventral striatum, which has been implicated in primary reward representations and expected outcomes following Pavlovian associative learning processes, and the dorsomedial striatum, which has been linked to cognitive control of instrumental responses and goal-directed behaviors (Gruber and McDonald, 2012), are two striatal areas of interest. These brain regions have been shown to elicit abnormal responses in individuals with various aggressive phenotypes (Crockett et al., 2013). Accordingly, increased striatal volumes in both forensic and community populations are associated with psychopathy (Cope et al., 2012; Korponay et al., 2017). Our group has conducted functional connectivity studies involving the striatum in relation to MAOA. In impulsive and violent patients with ASPD and high levels of psychopathy, there was a positive correlation with MAOA brain expression and functional connectivity of the superior ventral striatum with the dorsomedial PFC (Kolla et al., 2016). These are some of the first data that suggest that brain levels of MAOA can influence functional connectivity between brain areas that are involved in the regulation of aggressive behavior.

In summary, brain regions involved in learning/memory and emotional regulation modulate aggressive behavior. Changes in the structure and/or functioning of these regions likely increase aggressive behavior. Rosell and Siever (2015) suggested that the changes in functional connectivity are not always due to structural changes but could be a result of aberrant neuromodulation. Our finding that corticostriatal functional connectivity correlated with amounts of MAOA and also *Maoa* VNTR genotype supports this notion (Kolla et al., 2016, 2018).

### Interactions Among Parallel Learning and Memory Circuits Might Contribute to Aggression

It seems clear that individuals with aggressive phenotypes have alterations in various brain areas implicated in learning and memory and cognitive control functions. However, the implications of these neural changes on behavior might not be fully appreciated. Some, over the last few decades, have argued that mammalian learning and memory functions are organized into different neural circuits that operate in parallel. These complex networks are comprised of brain regions throughout the expanse of the brain including cortical, subcortical, and brainstem regions. There exists a significantly large body of evidence identifying six parallel neural circuits that seem to support different learning and memory functions, each with a central structure (amygdala, hippocampus, dorsolateral striatum, dorsomedial striatum, perirhinal cortex, and cerebellum). One interesting line of research has provided evidence that these parallel, functional networks provide coherent outputs *via* cooperative and/or competitive interactions. These dynamic interactions between these networks, in the average person, are seen as the key to normal brain functions and outputs like coherent thought processes, appropriate emotional reactions, good choices and actions, and even personality type (McDonald et al., 2004; Gruber and McDonald, 2012). We have used this theory to explain both normal and abnormal manifestations *via* a balance amongst these systems in the former and alterations of this balance between these systems through the influence of various factors including genetics, pre- and postnatal development, and experience. So, in essence, these parallel networks, the way they are organized and shaped, and the way they dynamically interact with one another, determine who we are and how we behave in particular situations. We refer to these different functional networks as memory-based behavioral systems because they have such a fundamental influence on behavior. For the expansion of these concepts, readers are encouraged to consult the following reviews: McDonald et al. (2004) and Gruber and McDonald (2012).

What does this theory of brain function tell us about aggression and individuals with aggressive phenotypes? It is possible that aggressive individuals with alterations in the size, function, or influence of brain regions associated with specific memory-based behavioral systems is two-fold. First, changes of one of these memory-based behavioral systems not only impairs the function of that system but it also can result in enhanced influence and control of other parallel systems not affected. For example, the memory-based behavioral systems centered on the amygdala are thought to be critical for tracking stimuli that predict the presence of biologically significant events (positive and negative) and does so *via* Pavlovian associative mechanisms (for review see, McDonald et al., 2017). This can result in difficulties in accurately predicting when negative events might occur. This uncertainty in stressful and fear-inducing situations could lead to aggressive outcomes. The loss of amygdalar influence can have another effect, a release of thought and behavior control from this important functional network and an enhancement of other circuits that may not provide the appropriate solution during the conflict. For example, the instrumental system centered on the dorsomedial striatum is under the influence of amygdalar representations as well as various prefrontal regions implicated in executive functions like inhibitory control. This system is more flexible and takes into account the context of the situation, previous experience (episodic memory), and the internal state of the individual to make choices about what response to make in a given situation (Gruber and McDonald, 2012). It is possible if the dorsomedial striatum is reduced in size and functional capacity that higher-order cognitive control will be diminished, resulting in a reduction of the ability to predict the consequences of certain instrumental responses in a particular situation (context, threat, etc.), leading to aggressive responses. Reduction in the influence of this memory-based behavioral circuit could also lead to the increased influence of the habit system. If these individuals have found aggressive behaviors to be reinforcing and/or rewarding, this could increase the habitual nature of these responses and would be released if the dorsomedial striatum was compromised. This could pose a distinct problem for individuals with aggressive phenotypes, because if instrumental responding is not under cognitive control, the habit system, a memory-based behavioral system centered on the dorsolateral striatum, could gain control of behavior and elicit inappropriate and aggressive responses that have been learned in these situations in the past and have been repeatedly reinforced.

Interactions between parallel learning and memory circuits have been posited to regulate both normal and abnormal behavior. Changes in these interactions between parallel learning and memory circuits provide an avenue for investigating how genetics and experiences can influence behavior. Imbalances in the interactions of some of these circuits likely contribute to the manifestation of aggressive behavior. Interestingly, circadian rhythm disruption affects behavior mediated by these same circuits. The next section will present behavioral and neurophysiological evidence that circadian rhythm disruption influences many of the mechanisms that underlie aggressive behavior.

### Circadian Rhythm Disruption

The previous section demonstrates that brain morphology, functional connectivity, and changes in interactions of neural circuits contribute to aggressive behavior. Circadian rhythm misalignment affects many of the same brain areas and circuits thought to underlie aggressive behavior.

First, circadian misalignment can inflict structural changes in the brain. In humans, chronic jetlag experienced by airline workers was associated with temporal lobe atrophy (Cho, 2001). The temporal lobe contains the hippocampus and its related cortical structures. As mentioned above, context is thought to be an important component of aggression. *Via* its connections with the OFC and ventral striatum, the hippocampus is involved in contextual representations that influence decision making (Zelinski et al., 2010; Gruber and McDonald, 2012; Trow et al., 2017). The hippocampus has been implicated in aggression, psychopathy, and ASPD with unilateral hippocampal atrophy and hypofunction reported (Raine et al., 1997, 2004; Critchley et al., 2000; Laakso et al., 2000, 2001). In rodents, hippocampal lesions impair the ability to accurately associate a context with an aversive stimulus (contextual fear conditioning; Phillips and LeDoux, 1992; Antoniadis and McDonald, 2000; Sutherland et al., 2010). Interestingly, psychopathy is also associated with impairments in fear conditioning (Flor et al., 2002; Birbaumer et al., 2005).

Structural changes after circadian misalignment have also been documented in animal models. We have found that circadian misalignment induces hippocampal atrophy when paired with a damaging agent (Gidyk et al., 2015). Further structural changes in response to circadian misalignment include changes in neuronal structure and function. In rodents, hippocampal neurogenesis has been shown to be decreased by various light manipulations that induce circadian dysfunction (Gibson et al., 2010; Fujioka et al., 2011). Structurally, dendritic length and density are reduced by circadian disruption in the medial PFC and hippocampus (Bedrosian et al., 2011; Karatsoreos et al., 2011; Fonken et al., 2012).

Second, neuromodulators are also affected by circadian rhythm disruption. Corticosterone, which is the rodent analog of cortisol in humans, can be elevated or phase advanced during circadian rhythm disruption (Gibson et al., 2010; Barclay et al., 2012; Deibel et al., 2014). The relationship between cortisol and testosterone plays a role in aggressive behavior; however, the nature of this relationship varies depending on age, gender, and degree of aggression or psychopathy (Rosell and Siever, 2015). In terms of neurotransmission, Bedrosian and Nelson (2017) suggest that as *Maoa* expression is oscillatory and regulated by clock genes, it is likely that environmental circadian rhythm disruption affects monoamine neurotransmitters. With this in mind, in rodents, depending on the experimental lighting schedule, 5-HT, DA, and BDNF can all be affected by environmental circadian rhythm disruption paradigms (Shieh et al., 1997; Matsumura et al., 2015; Ikeno and Yan, 2016). As mentioned previously, these neurotransmitter systems are involved in the modulation of aggressive behavior.

Finally, environmental manipulations of the light-dark cycle induce learning and memory impairments in tasks mediated by brain areas and circuits involved in aggression. Shifts of the light-dark cycle or non-24-h days can be used to induce circadian misalignment (Zelinski et al., 2014a; Baron and Reid, 2015). Various paradigms, in rodents, induce memory impairments in tasks that involve the hippocampus, amygdala, medial PFC, and striatum (Devan et al., 2001; Craig and McDonald, 2008; Ruby et al., 2008; Gibson et al., 2010; Loh et al., 2010; McDonald et al., 2013; Zelinski et al., 2013, 2014b; Deibel et al., 2014; Fernandez et al., 2014). Given that circadian rhythm misalignment induces dysfunction in the brain areas and circuits involved in aggression, it is possible that in these models aggression in also elevated. To our knowledge aggression has never been assessed in rodents subjected to environmental circadian rhythm disruption.

Circadian rhythm disruption affects many of the same brain regions, neuromodulators, and circuits that modulate aggressive behavior. It is possible that circadian rhythm disruption is eliciting some of the changes in interactions among learning and memory circuits that we hypothesized contribute to aggression.

## Section 4: Circadian Misalignment and Aggression

Hopefully, we have laid out a pathway in which circadian misalignment could affect aggressive behavior by influencing overlapping genetics and neural circuitry. However, the strongest piece of evidence to support this claim would be data that circadian misalignment is associated with increased aggression.

Circadian misalignment is thought to be a mechanism for the effects of chronotype on health and disease; however, as mentioned above, very few studies actually measure circadian misalignment with objective or subjective measures (Baron and Reid, 2015). Also, as the majority of the studies described are correlational, the direction of the true relationship is unknown. It is possible that the behavior or disease state is influencing circadian rhythms (Baron and Reid, 2015; Hood and Amir, 2018). Recently, with the finding that late chronotype is associated with antisocial and borderline personality disorders (Fleischer et al., 2012; Jonason et al., 2013; Schlarb et al., 2014; Rahafar et al., 2017), chronotype has been thought to contribute to aggressive behavior.

While this is a burgeoning area of research, few studies have investigated chronotype and aggression directly. Several studies have investigated chronotype and affective temperaments, which are traits that are thought to predispose one to affective disorders, such as depression and bipolar disorder (Park et al., 2015; Chrobak et al., 2018). Park et al. (2015) found an association between eveningness and traits related to anger, whereas Chrobak et al. (2018) did not detect this association. However, aggression is only indirectly measured in the temperament evaluation of Memphis, Pisa and SanDiego-Autoquestionnaire scale, which was used for both of the aforementioned studies. Some studies have, indeed, measured chronotype and aggression more directly. Readers are encouraged to refer to Schlarb et al. (2014) for a systematic review of chronotype and aggression during childhood and adolescence. Of the 13 studies that met their inclusion criteria, only one did not find a relationship between eveningness and increased aggression (Schlarb et al., 2014). We will focus on several recent studies that have measured chronotype in young adults and adults.

Aspects of reactive aggression has been associated with a late chronotype. Hostility, anger, and impulsivity were increased in those with an evening chronotype (Randler and Vollmer, 2013; Hwang et al., 2016). Interestingly, social jetlag was positively correlated with physical aggression (Randler and Vollmer, 2013). That social jetlag predicted physical aggression (Randler and Vollmer, 2013) provides initial evidence that circadian misalignment can elicit aggression. It should be noted however that having a late chronotype does not always infer circadian rhythm disruption. For example, in this study and another that investigated participants with ADHD, social jetlag and chronotype differed in their explanatory power of the dependent variable (Randler and Vollmer, 2013; McGowan et al., 2016).

While factors related to reactive aggression are associated with an evening chronotype, aggression is a very complex behavior that can present in different forms depending on personality traits or psychiatric illness. Some have thought that a subset of humans have evolved to favor more nocturnal activity patterns (Jonason et al., 2013). Nocturnality might be advantageous because less people are active during the night, there is decreased light, and cognition of morning types is compromised (Jonason et al., 2013). It was hypothesized that those with high dark triad personality traits—psychopathy, narcissism, and Machiavellianism—might favor eveningness. Machiavellianism, secondary psychopathy, and exploitive narcissism were correlated with eveningness (Jonason et al., 2013). Secondary psychopathy is characterized by more emotional reactivity and psychological turmoil than primary psychopathy (Vaughn et al., 2009). Except for narcissism, the finding that those scoring higher on the dark triad traits were more prone to eveningness has been replicated (Rahafar et al., 2017). Interestingly, mediation analyses determined that eveningness explained the variance in the effect of gender on the dark triad traits, although this effect was small (Rahafar et al., 2017). These data further confirm that dark triad traits are associated with eveningness and also that eveningness could possibly mediate the tendency for males to score higher in the dark triad traits.

The association of eveningness with increased dark triad traits suggests that factors related to proactive aggression are associated with eveningness. Some recent studies investigate the relationship of chronotype with a new form of proactive aggression. Cyberbullying involves remote digital harassment or mistreatment of people who cannot defend themselves (İçellioğlu and Özden, 2014; Kircaburun and Tosuntaş, 2018; Tosuntaş et al., 2020). Recently, eveningness was found to be associated with the greater commission of cyberbullying by young adults (Kircaburun and Tosuntaş, 2018) and adolescents (Tosuntaş et al., 2020). In concert with the dark triad data, the cyberbullying data suggests that proactive aggression is associated with chronotype.

The studies presented in this section demonstrate that factors contributing to both reactive and proactive aggression are associated with an evening preference. It is important to note that these studies measure chronotype primarily with subjective questionnaires. The one study above that used a more quantitative measuring tool (MCTQ) in conjunction with a subjective questionnaire (Randler and Vollmer, 2013), found that the results depended on the tool used. Another caveat to consider is that the majority of the studies that have measured chronotype have sampled the general population. This observation could explain why most studies report relatively small effect sizes (Jonason et al., 2013; Rahafar et al., 2017). To our knowledge, chronotype has not been assessed in people with a protracted history of violence, such as individuals with ASPD or psychopathy. We expect the finding of increased aggression in evening types to be even more pronounced in these individuals. While the evidence suggests that aggression is related to eveningness, the direction and mechanisms for this relationship are unknown. Several studies in this section suggest that circadian misalignment might be a possible mechanism for the relationship between eveningness and increased aggression.

## Conclusions

With society’s reliance on shiftwork and artificial light, it has never been more important to uncover the effects of circadian rhythm disruption on the brain and body. Based on recent behavioral, genetic, and neurobiological research, we suggest that circadian rhythm disruption contributes to aggressive behavior. Behaviorally, recent studies link an evening preference to aggression, and we argue that an evening preference is often associated with mild yet chronic circadian misalignment. Interestingly, genes that are affected by circadian misalignment also contribute to the expression of aggressive behavior. It is possible that circadian misalignment elicited by either genetic variation and/or the environment has a downstream effect on systems that underlie aggression, such as the dopaminergic and serotonergic pathways. In terms of the neurobiology, circadian misalignment induces similar changes in many of the same structures thought to mediate aggression. We hypothesize that changes in the interactions among these circuits are contributing to the manifestation of aggressive behavior. It is important to note that circadian misalignment and aggression is a relatively untouched area of research. It is our hope that the literature reviewed and hypotheses presented in this article serve to spur on future research in this important and topical area of research.

## Author Contributions

SD designed the review and wrote the manuscript. RM contributed to the neurobiology section. NK wrote and edited the manuscript.

## Conflict of Interest

The authors declare that the research was conducted in the absence of any commercial or financial relationships that could be construed as a potential conflict of interest.
